# Significant differences between serum CRP levels in children in different categories of physical activity: the PLAY study

**DOI:** 10.5830/CVJA-2010-005

**Published:** 2010-12

**Authors:** Berna Harmse, H Salome Kruger

**Affiliations:** School of Physiology, Nutrition and Consumer Science, North-West University, Potchefstroom, South Africa; School of Physiology, Nutrition and Consumer Science, North-West University, Potchefstroom, South Africa

**Keywords:** C-reactive protein, inflammation, physical activity, adolescent, metabolic syndrome, body composition

## Abstract

**Summary:**

Low-grade systemic inflammation is emerging as a component of the metabolic syndrome. The purpose of this study was to assess the association between serum C-reactive protein (CRP), physical activity and body composition in 193 black children aged 13 to 18 years from a South African township. Demographic information and anthropometric measurements were taken, and fasting blood samples were analysed for high-sensitivity serum CRP. Body fat was measured by air displacement plethysmography.

There was a trend towards higher serum CRP in the boys with a higher percentage body fat. After multiple regression analyses, waist circumference in the girls was significantly associated with serum CRP. In the boys, there was an inverse correlation between percentage body fat and fitness, and between fitness and serum CRP. Significant differences were found between serum CRP in the different physical activity categories, with lower serum CRP in the girls in the higher physical activity group. Obesity should be prevented in South African children by encouraging physical activity.

## Summary

Obesity is currently the most common and costly nutritional problem in developed countries,^1^ with 10% of the world’s school-aged children estimated to be overweight.^2^ The prevalence of overweight is rising significantly in most parts of the world, especially in economically developed regions. This rapid increase implicates environmental rather than genetic factors,^2^ although one cannot exclude the interaction between genes and the environment.^3^

The metabolic syndrome (MS), a cluster of five biological markers that together predict the development of cardiovascular disease and type 2 diabetes, is now increasingly emerging among children and adolescents.^4^ These markers are hypertension, insulin resistance, central adiposity, hypertriglyceridaemia and decreased high-density lipoprotein cholesterol. Low-grade systemic inflammation is increasingly being observed as a significant component of the MS.5 Various cytokines and chemical messengers, which induce their effects individually or in interaction with each other, constitute the main regulators of the inflammatory process.^5^

Young people in lower-income families are particularly vulnerable to obesity because of poor diet and limited opportunities for physical activity. In developing countries, obesity among the youth is most prevalent in wealthier sections of the population, but it is also rising among the urban poor in these countries, possibly due to their exposure to westernised diets, coinciding with a history of under-nutrition.^2^ Lobstein *et al*.^2^ emphasised that children from socio-economically deprived environments in most western societies have a greater risk of obesity than children from more affluent groups.

Adolescence is an important period during development, and significant somatic growth and maturation are evident during this time. The pubertal growth spurt is associated with significant changes in body composition, where girls tend to accumulate more fat than boys.^4^ Adolescence is one of the most vulnerable periods for the development of overweight and obesity.^2^ Although the mechanism is unclear, it is possible that fat distribution patterns established during adolescence play a role.^2^ The maximum body mass index attained at post-pubescence is strongly associated with the degree of fatness in adulthood.^3^

South Africa is a developing country with both under- and over-nutrition. The prevalence of obesity is high among black women, whereas it is low in children.^6^ Stunting is a very common nutritional disorder in South Africa and local research has shown that there may be a link between stunting and the development of overweight or obesity.^7^ According to Monyeki *et al*.,^8^ obesity is not only common in South African women, but also in female adolescents. In 2002 the first South African National Youth Risk Behaviour survey stated that the prevalence of overweight among secondary school learners was 17% and obesity 4%.^9^ These data also showed that the co-existence of stunting and being overweight is a public health problem among adolescents in South Africa.^9^

Obesity during childhood seems to increase the risk of subsequent morbidity, whether obesity persists into adulthood or not, with obese children being at an increased risk of metabolic and cardiovascular disorders later in life.^10^ Recent South African studies indicate that measures of overweight were positively associated with increased blood pressure,^11^ plasminogen activator inhibitor-1 activity, plasma fibrinogen and the thrombin–anti-thrombin complex,^12^ as well as higher fasting plasma insulin and leptin levels and insulin resistance. These possibly increase the risk for cardiovascular disease.^13^ No South African studies on body composition and low-grade inflammation in children or adolescents could be found in the literature.

C-reactive protein (CRP), synthesised in the hepatocytes, is an acute-phase reactant that responds non-specifically to infections, immuno-inflammatory diseases and malignancies.^14^ CRP is also a surrogate marker for interleukin (IL)-6 activity and is proven to predict the development of type 2 diabetes and mortality.^15^ Levels of CRP are usually low or undetected in healthy subjects, but they increase up to 100 times during acute illness or inflammation.^16^ In the absence of infection, elevations of CRP levels, generally below 10 mg/l, are associated with an increased risk of the development of atherosclerotic cardiovascular disease.

In recent years CRP values, as measured by a high-sensitivity assay (hs-CRP), have been recognised as a useful and sensitive predictor of the future risk of myocardial infarction and stroke.^17^ Pepys and Hirschfield^14^ noted that CRP values cannot be used diagnostically, but should be interpreted with full knowledge of all other clinical and pathological results. Upon interpretation of CRP values, low-risk inflammation is defined as a level less than 1 mg/l, average risk is 1.0–3.0 mg/l, and high-risk values are 3–10 mg/l.^18^

There is a link between basal inflammation, MS and obesity. The release of IL-6 from the visceral adipose tissue may induce low-grade systemic inflammation in subjects with increased body fat, resulting in clinically raised CRP levels in obese adults.^14^,^18^ However, the limited number of studies on children causes uncertainty about the clinical significance of inflammation in overweight children.^4^,^5^,^16^,^17^,^19^

Body fatness and central body fat distribution are related to an adverse risk profile in the youth.^17^ A higher degree of cardiorespiratory fitness has been shown to relate to a healthier metabolic profile in children. Isasi *et al*.^19^ showed in a study in the USA that CRP levels did not differ between boys and girls and fitness level was inversely correlated to CRP in the boys. There are, however, few data about the association between obesity, physical activity and serum CRP concentrations in black South African children.

Early assessment of the inflammatory status of South African children could be useful to decrease CVD risks, particularly in individuals at intermediate risk, such as overweight or physically inactive children. The purpose of this study was to assess the association between serum C-reactive protein and physical activity, as well as body composition in 193 black high-school children aged 13 to 18 years from a township in South Africa.

## Methods

This study was performed as part of the PLAY study (PhysicaL Activity in the Young) on the effects of physical activity in children. For the purposes of this study, baseline data were used for cross-sectional analysis.

The PLAY study was approved by the Ethics Committee of the North West University (Potchefstroom campus). All grade 9 children (13–18 years old) from two secondary schools, situated in a low-income area in Potchefstroom in the North West Province of South Africa, were provided with a permission form to be signed by their parents before inclusion in the study. Permission was also obtained from the school principals and consent from the children. In total 193 boys and girls were available for inclusion.

The children were picked up at the schools and brought to the University metabolic unit on the study days. They started at the blood station, where fasting blood samples were taken. Thereafter body composition (height, weight, waist circumference, skin folds, and body volume and density) was measured. The children then received food and proceeded to the questionnaire stations (demographic, Tanner and PDPAR).

Data regarding age, gender, home language, socio-economic status, housing, accessibility to water and electricity, smoking status and general health of the children, as well as educational level and occupation of the parents/caregivers were obtained by individual interviews, performed by fieldworkers in each subject’s language of preference.

## Body composition and anthropometry

The subjects were measured and weighed in their underwear by trained postgraduate biokinetics students according to standard ISAK (International Society for the Advancement of Kinanthropometry) methods.^20^ The height (cm) of the subjects was taken with a vertical stadiometer to the nearest 0.1 cm. Body weight was measured to the nearest 0.1 kg on an electronic scale (Precision Health scale, A & D Co, Saitama, Japan). The scale was calibrated with a 10-kg standardised weight.

Anthropometrical nutritional status was defined by Centers for Disease Control (CDC) *z*-scores^21^ and international body mass index (BMI)-for-age cut-off points.^22^ A flexible steel measuring tape (Lufkin, Cooper Tools, Apex, NC, USA) was used for measuring waist and hip circumferences to the nearest 0.1 cm and waist:hip ratio (WHR) was calculated by dividing waist circumference by hip circumference. Skin folds were measured using a John Bull® (British Indicators, London, UK) skin-fold calliper to the nearest 0.1 mm of the triceps, sub-scapula, medial calf, abdominal and supra-spinal skin folds. Landmarks were drawn first where-after post-graduate biokinetics students with a level 2 anthropometic qualification did the measurements.

The children’s body composition (percentage muscle and fat) was measured by air-displacement plethysmography (BOD-POD, Life Measurement Inc, Concord, CA), which was calibrated at the start of each day’s measurements with a cylinder of standardised volume. The BOD-POD system uses the principle of whole-body densitometry to obtain the amount of fat and lean body mass in the body. For accuracy of measurement, the subjects wore tight-fitting clothing and a swimming cap, removed all jewellery and emptied the bladder before testing. Minimal movement of the subjects within the BOP-POD was ensured. The subjects were shown how to use the thoracic gas volume tubes and a measurement was taken to compensate for lung volume.

Body volume (litre) was measured with the BOD-POD, using the ratio between pressure and volume, as explained by Boyle’s law. The ratio is used to calculate the unknown volume by measuring the pressure directly. The pressure in both chambers reacts immediately and the difference in pressure represents the relative volume of air in each chamber.

Body density (Db) was calculated by dividing the body’s mass by the volume, using the Siri equation.^23^ From Db, the fat percentage, fat mass, lean body mass and lung volume could be obtained. Two measurements were taken and the aggregate was used.

Trained professionals in private rooms used the Tanner staging scale questionnaire to estimate the physical maturity of the boys and girls.^24^

## Blood samples and biochemistry

Registered nurse practitioners obtained fasting blood samples from the subjects. The vena cephalica was used to draw 20 ml venous blood for the preparation of EDTA plasma and serum. Blood was centrifuged for 15 minutes at 4°C and 2 000 × *g* for serum and plasma preparations, divided into Eppendorff tubes and frozen at –84°C until the analyses were done.

High sensitivity C-reactive protein (hs-CRP) was measured by immuno-nephelometry (Cardiophase hsCRP, Dade Behring, 2004) at an accredited laboratory (Ampath Laboratories, Pretoria). Control serum with a concentration of 13.5 mg/l was used as an external standard. The mean concentration of the controls was 13.53 mg/l, with a range of 13.0–14.3 mg/l and a coefficient of variation of 8.6%.

## Physical activity and fitness

Trained field workers used the Previous day physical activity recall questionnaire (PDPAR) to obtain information regarding subjects’ physical activity of the previous day (24 hours) and a 24-hour day the previous weekend.^25^

Cardiovascular fitness was determined by means of the bleep test. Participants had to run a 20-m distance, timed by a metronome. The running pace increased with each level (levels 1 to 6). When a participant could no longer complete the distance within the allocated time, the test was terminated and the corresponding level of fitness was recorded. The levels were converted to indirect maximum rate of oxygen consumption (VO_2_ max) values using a conversion calculator.^26^

## Statistical analysis

The Statistica computer data analysis software system from Statsoft, Inc (2004), version 7 was used to analyse data. CRP data were not normally distributed and were transformed logarithmically. Descriptive statistics were used to describe the characteristics of the subjects. The Mann-Whitney *U*-test, as well as Kruskall-Wallis tests were performed to assess differences between serum CRP levels of the children in the different categories of habitual physical activity, as well as to compare serum CRP levels of children with normal or low percentage body fat with those with high body fat percentages. Spearman correlation was performed to assess the correlation between body composition variables, serum CRP (log transformed), reported physical activity and fitness of the children.

## Results

The sample comprised black children living in a poor socio-economic setting. The type of housing was generally of galvanized zinc or brick with partial water and electricity supplies. Subjects in the two schools were in a similar growth phase and socio-economic status, with comparable eating habits and physical activity levels. Only 5.7% of the children admitted to smoking, and one was a girl. The median age of starting to smoke was 15 years (interquartile range 13–17) and they smoked a median of six cigarettes per day (interquartile range 2–10).

The serum CRP concentrations of the children ranged between < 0.2 and 39.8 mg/l. Three girls and three boys with high serum CRP concentrations, above 10 mg/l, were excluded from further analysis because this could have been due to acute inflammation.^16^,^17^ Most of the children had serum CRP concentrations within the normal range (< 3 mg/l), and only a few had raised levels. The children’s serum CRP concentrations were then compared with regard to habitual physical activity level and body fat percentage. Because the data were not normally distributed, median and interquartile ranges were also calculated.

The descriptive statistics of girls and boys per category of habitual physical activity are shown in Table 1. For the purpose of this study, ‘inactive’ was defined as children with a score of 1, moderately active was a score of 2, and ‘most active’ was a score of 3, according to the coding for the PDPAR, as proposed by Weston *et al*.^25^

**Table 1.** Descriptive Statistics Of Children Per Category Of Habitual Physical Activity (Mean ± SD/Median, Interquartile Range^#^)

*Variable*	n*	*Inactive*	*n*	*Moderately active*	*n*	*Most active*
Girls						
BMI (kg/m^2^)	59	20.43 ± 2.94	35	19.96 ± 3.60	10	20.22 ± 3.02
Bleep test score	58	3.27 ± 0.90	37	3.64 ± 1.31	9	4.47 ± 0.93
CRP (mg/l)^#^	58	0.33 (0.15–0.94)	38	0.46 (0.26–1.75)	10	0.22 (0.21–0.26)
WC (cm)	60	64.54 ± 5.90	38	63.82 ± 6.42	10	63.35 ± 4.13
TSF (mm)	60	16.60 ± 5.91	38	14.50 ± 4.47	10	14.12 ± 5.13
Boys						
BMI (kg/m^2^)	18	19.35 ± 2.58	34	18.56 ± 2.43	23	19.51 ± 2.92
Bleep test score	18	6.43 ± 2.49	33	6.2 ± 1.75	23	7.00 ± 1.91
CRP (mg/l)^#^	17	0.19 (0.15–0.38)	32	0.59 (0.28–1.06)	21	0.31 (0.16–0.95)
WC (cm)	18	66.42 ± 4.95	33	64.81 ± 5.90	22	66.41 ± 5.60
TSF (mm)	18	9.12 ± 4.09	33	8.41 ± 4.11	22	9.36 ± 4.94

BMI: body mass index; CRP: C-reactive protein; WC: waist circumference; TSF: triceps skin fold. *Number of subjects differs due to missing data.

There were significant differences between serum CRP concentrations for the three physical activity categories of girls and boys. The Kruskall-Wallis value for the difference in log CRP in the girls was *H* = 7.33 (*p* = 0.025) and for the boys *H* = 7.5 (*p* = 0.02). The difference in CRP between activity groups showed lower median serum CRP with higher physical activity in the girls, as seen in Table 1. In the boys there was no clear trend, with the highest median serum CRP in those with a moderate physical activity level (Table 1).

With correlation analyses, in the girls there was a significant positive correlation between the reported physical activity level and their bleep test results (number of laps completed). The bleep test result also correlated negatively with all body composition variables indicating obesity (BMI, waist circumference, skin folds, body fat percentage).

In the boys, a significant negative correlation was found between the bleep test result and body fat percentage, as well as skin fold thickness, but no correlation between bleep test result and reported physical activity was found (Table 2). There was, however, a significant negative correlation between serum CRP and the number of laps completed by the boys, as well as calculated VO_2_ max, indicating an inverse association between fitness and serum CRP. In the girls, the association was in the same direction, but not statistically significant.

**Table 2.** Pearson Partial Correlations For Boys And Girls Adjusted For Age And Smoking

*Parameters*	*Boys*	n	p	*Girls*	n	p
BMI and log CRP	0.01	72*	NS	0.21	112	0.04
Waist circumference and log CRP	–0.03	69	NS	0.22	112	0.03
Triceps skin fold and log CRP	0.10	69	NS	0.15	112	NS
Sub-scapular skin fold and log CRP	0.16	69	NS	0.20	112	0.05
Supra-spinal skin fold and log CRP	0.12	69	NS	0.28	112	0.004
Abdominal skin fold and log CRP	0.13	66	NS	0.21	112	0.03
Body fat percentage and log CRP	0.16	53	NS	0.14	106	NS
Bleep test laps completed and log CRP	–0.30	71	0.012	–0.15	109	NS
Bleep test laps completed and PDPAR score	0.06	112	NS	0.22	131	0.01
Bleep test laps completed and body fat percentage	–0.26	81	0.02	–0.33	128	< 0.0001

*Number of subjects differs due to missing data.

Comparison of girls with a body fat percentage above 25% (over-fat) with lean girls showed the lean group had a median serum CRP of 0.30 mg/l (interquartile range 0.15–0.9) and the over-fat group a median of 0.34 mg/l (interquartile range 0.2–1.0) (Table 3). There was therefore no significant difference in serum CRP levels between the two groups.

**Table 3.** Descriptive Statistics For Girls With High Body Fat Percentage Versus Girls With Normal Body Fat Percentage

*Variable*	*Body fat % > 25% (n = 35)*		*Body fat % > 25% (n = 101)*	
	*Mean/number*	*Standard deviation/%*	*Mean/number*	*Standard deviation/%*
Age (years)	15.43	1.24	15.55	1.41
Weight (kg)^#^	43.98	5.88	51.80	8.52
Height (cm)	155.6	0.04	155.0	6.67
BMI (kg/m^2^)^#^	18.12	2.11	21.53	3.09
Waist circumference (cm)	60.55	4.24	66.28	5.78
WHR	0.75	0.04	0.76	0.05
Triceps skin fold (mm)^#^	10.99	3.87	18.11	5.93
Sub-scapular skin fold (mm)^#^	9.00	2.95	13.99	5.51
Abdominal skin fold (mm)^#^	12.82	4.15	22.09	7.11
Fat %^#^	20.60	4.64	32.06	4.74
Muscle mass (kg)	33.57	5.16	34.66	5.28
Tanner stage 1* (14 years)	2	5.7%	0	0
Tanner stage 2* (14.4 ± 0.6, 13–15 years)	5	14.3%	5	4.9%
Tanner stage 3* (15.2 ± 1.2, 14–17 years)	10	28.6%	46	45.5%
Tanner stage 4* (15.7 ± 1.2, 14–18 years)	16	45.7%	43	42.6%
Tanner stage 5* (16.6 ± 1.4, 15–18 years)	2	5.7%	7	6.9%
	*Median*	*Interquartile range*	*Median*	*Interquartile range*
Serum CRP (mg/l)	0.30	0.15–0.91	0.34	0.20–1.23

Differentiated on account of fat percentage, girls with fat percentages > 25% were classified over-fat, according to Lohman.^28^ *Tanner stage based on breast/genital development stage (1 = no development, 5 = mature)^21^ with age, mean ± SD (range), ^#^significant differences between groups. BMI: body mass index; CRP: serum C-reactive protein concentration; WC: waist circumference; TSF: triceps skin fold.

Comparison of the data for serum CRP in the boys showed a similar result, where the boys with higher body fat percentages (> 20%) had a median serum CRP value of 0.5 mg/l (interquartile range 0.2–1.9) and the lean boys a median of 0.32 mg/l (interquartile range 0.15–0.8). The Mann-Whitney *U*-test for the difference in CRP levels in boys was *z* = 1.39 (*p* = 0.16), indicating no significant difference, but a trend towards higher serum CRP concentrations in the boys with higher percentages of body fat.

Multiple regression analyses were performed with age, cigarette smoking, body fat percentage, waist circumference and physical activity level as predictor variables and serum CRP (log transformed) as dependent variable. Children with a serum CRP level > 10 mg/l were excluded from these analyses. For the boys, no variable was significantly associated with serum CRP. In the girls’ group, age and waist circumference were statistically significantly associated with serum CRP, although the adjusted *R*^2^ was small (adjusted *R*^2^ = 0.08, *p* = 0.007).

## Discussion

The purpose of this study was to assess the association between serum CRP levels, physical activity and body composition in black adolescents in two township schools. The most active girls had the lowest serum CRP values, similar to the findings of Isasi *et al*.,^19^ with a significant difference between the CRP values of active girls versus girls with low habitual physical activity. The girls probably reported their physical activity more accurately than the boys because their physical activity correlated significantly with their bleep test scores, which measures actual fitness.^27^

The results for the boys, however, are difficult to explain because the boys in the inactive group had lower serum CRP values than those in the most active group. A possible reason may be that most boys were relatively fit and active and that other factors were more important determinants of their serum CRP concentrations. Alternatively, the boys may not have reported their physical activity as accurately as the girls, or some of the boys may recently have become inactive. Another reason may have been the absence of organised physical activity in the township, but that the boys participated in active play on an irregular basis and could keep fit in this way.

The boys’ group on the whole, however, (inactive plus active) showed trends as expected regarding physical activity and CRP levels. The fitness level of the boys, measured by number of laps completed in the bleep test, correlated negatively with body fat percentage and abdominal skin fold, as well as with serum CRP levels. If fitness is taken as a measure of habitual physical activity, this was an indication of an inverse association between physical activity and serum CRP levels in the boys.

As was expected, physical activity was higher in the boys than the girls.^28^ Studies have shown a negative correlation between physical activity and body fatness.^1^,^2^ It has been suggested that decreased physical activity or increasing inactivity is probably the main factor accounting for the reduction in total energy expenditure, leading to a positive energy balance and increased prevalence of obesity.^1^,^2^ Egger and Swinburn^29^ concluded that even incidental activity can increase energy expenditure, and intensity of activity also plays a role. Such incidental activity may have played a role in the relatively low number of boys with a body fat percentage above 20% in the study sample.

The CRP values of some of the children were above normal concentrations. On interpretation of CRP values, low risk is defined as a level less than 1 mg/l, average risk is 1.0–3.0 mg/l and high risk is values of 3–10 mg/l.^18^ In this study, when subjects with markedly increased CRP levels (> 10 mg/l) were excluded, a significant association was observed between physical activity and CRP levels in the girls and between fitness and CRP levels in the boys. The relevance of such findings in adolescents is useful because CRP level is proven to predict the development of type 2 diabetes and mortality.^5^,^30^ Future risk can therefore be assessed in adolescents.

Smoking is a well-known predictor of high serum CRP levels.^19^ Only 5.7% of the children admitted to smoking and among those who smoked, only one was a girl. Adjustment for smoking in the correlation analyses did not, however, make any difference to previously non-significant correlations. In this low-income setting, children could probably not afford to smoke regularly.

A larger number of girls in comparison with boys were classified as over-fat. Only 15 out of a total of 85 boys had a fat percentage higher than the cut-off of 20%. Out of 134 girls, only 35 had a fat percentage equal to or below 25%, with 101 girls having fat percentages above this norm.^31^ According to Monyeki *et al.*,^8^ obesity is not only common in South African women, but also in female adolescents. Adolescence is one of the most vulnerable periods for the development of overweight and obesity.^2^,^2^

The girls in our study live in a low socio-economic environment, and it is possible that low physical activity and consequently a low fitness level played a role in the development of their high body fat percentages. Almost 50% of the girls were in Tanner stages 4 and 5 and 74% of the girls had a body fat percentage above 25%, compared to 71% of the boys in Tanner stages 4 and 5, but only 18% of them had a body fat percentage above 20%.

With regard to skin folds, the greatest difference between the group of boys with a body fat percentage above 20% and the group with a normal body fat percentage was for the abdominal skin fold. This indicates that fat accumulation in this group of boys was mainly in the abdominal area.

Tables 3 and 4 show the differences in body composition and fat distribution between the boys and girls. The girls had greater skin fold thicknesses than the boys, but smaller WHR, indicating more abdominal fat distribution in the boys, compared to more peripheral fat distribution in the girls. This difference in body composition between the boys and girls is probably the main reason for the gender differences found in the associations between body composition and serum CRP levels. It is well-known that adolescent girls generally have a larger proportion of body mass as fat, and are more likely to deposit fat subcutaneously and on their hips, whereas adolescent boys are more likely to have more muscle mass and they deposit fat in the abdominal region, as shown in Tables 4 and 5. Oestrogen levels appear to underlie many of these gender differences.^32^

**Table 4.** Descriptive Statistics For Boys With High Body Fat Percentage Versus Boys With Normal Body Fat Percentage

*Variable*	*Body fat % > 20% (n = 70)*		*Body fat % > 20% (n = 15)*	
	*Mean/number*	*Standard deviation/%*	*Mean/number*	*Standard deviation/%*
Age (years)	15.88	1.56	15.65	1.44
Weight (kg)^#^	47.96	8.55	53.80	14.46
Height (cm)	161.31	8.66	159.51	10.30
BMI (kg/m^2^)^#^	18.32	2.28	20.87	3.69
Waist circumference (cm)	65.11	4.94	69.60	7.12
WHR	0.84	0.06	0.83	0.04
Triceps skin fold (mm)^#^	7.74	2.60	14.14	6.28
Sub-scapular skin fold (mm)^#^	7.18	1.82	12.20	8.18
Abdominal skin fold (mm)^#^	9.50	4.33	21.32	11.01
Fat %^#^	16.16	4.21	29.62	4.23
Muscle mass (kg)	40.45	7.49	37.99	10.50
Tanner stage 1* (–)	0	0	0	0
Tanner stage 2* (14.9 ± 0.7, 13–15 years)	6	8.6%	1	6.7%
Tanner stage 3* (15.0 ± 1.2, 13–17 years)	14	20%	4	26.6%
Tanner stage 4* (16.0 ± 1.3, 14–18 years)	44	62.8%	9	60%
Tanner stage 5* (16.8 ± 1.0, 15–18 years)	6	8.6%	1	6.7%
	*Median*	*Interquartile range*	*Median*	*Interquartile range*
CRP (mg/l)	0.39	0.15–0.90	0.59	0.20–1.65

Differentiated on account of fat percentage, boys with fat percentages > 20% were classified over- fat, according to Lohman.^28^ *Tanner stage based on genital development stage (1 = no development, 5 = mature)^21^ with age, mean ± SD (range), ^#^significant differences between groups. BMI: body mass index; CRP: serum C-reactive protein concentration; WC: waist circumference; TSF: triceps skin fold.

**Table 5.** Differences Between Anthropometric Variables And Correlation Between Percentage Body Fat And Anthropometric Variables In Boys And Girls With A High Body Fat Percentage Versus Boys And Girls With A Normal Body Fat Percentage

Correlation coefficient (level of significance) for the correlation between percentage body fat and skin-fold thickness				
*Variable*	*Boys*		*Girls*	
	*Body fat % > 20% (n = 70)*	*Body fat % > 20% (n = 15)*	*Body fat % > 20% (n = 35)*	*Body fat % > 20% (n = 101)*
Triceps skin fold (mm)	0.16 (NS)	0.63 (*p* = 0.001)	0.33 (NS)	0.63 (*p* = 0.001)
Sub-scapular (mm)	–0.03 (NS)	0.67 (*p* < 0.0001)	0.25 (NS)	0.67 (*p* < 0.0001)
Abdominal (mm)	0.28 (*p* = 0.04)	0.68 (*p* < 0.0001)	0.37 (*p* = 0.05)	0.68 (*p* < 0.0001)
Percentage difference between groups of boys and girls				
	*Boys with body fat % > 20% compared with boys with body fat % > 20%*		*Girls with body fat % > 25% compared with girls with body fat % > 25%*	
Body weight (kg)	12.13%		17.78%	
Waist circumference (mm)	6.9%		9.5%	
Percentage body fat	83.3%		55.6%	

The release of IL-6 from the visceral adipose tissue may induce low-grade systemic inflammation in subjects with increased body fat. This may explain the association between BMI and CRP levels.^16^,^19^ Although in this study there was no statistically significant difference between the serum CRP levels of children with a normal body fat percentage versus those with a high body fat percentage, serum CRP values correlated positively with BMI, waist circumference and truncal skin folds in the girls. Among the boys, no significant positive correlation was found, but there was a trend towards an association in the same direction. Using multiple regressions with both physical activity and body composition variables, waist circumference was positively associated with serum CRP levels in the girls.

Also in line with previous reports, there was a trend towards an inverse correlation between fitness and percentage body fat in the boys in this study.^1^,^28^ Obesity and central body fat distribution are related to an adverse risk profile in the youth, and reports suggest that physical activity exerts a positive effect on risk factors for chronic disease. A higher degree of cardio-respiratory fitness has also been shown to relate to a healthier metabolic profile in children.^10^

Rapid urbanisation and westernisation are becoming the norm in South Africa and this may be causal to the notion that overweight in childhood could become a public health issue.^8^,^9^ Lobstein *et al.*^2^ and Simon *et al.*^33^ emphasised that children from socio-economically deprived environments in most westernised societies, such as the subjects of low socio-economic status in this study, have a greater risk of obesity than those from more affluent groups.

Findings in this study have broad implications, especially with regard to the link between body composition, cardiovascular risk and the metabolic syndrome. Many obese children, and especially adolescents, tend to become obese or overweight as adults and it has been suggested that 33% of adult obesity starts in childhood.^1^,^3^ Obesity during childhood seems to increase the risk of subsequent morbidity, whether or not obesity persists into adulthood,^1^,^34^ with obese children being at an increased risk of metabolic and cardiovascular disorders later in life.^3^,^5^

Limitations of this study include the fact that the age range of children recruited from one high school class varied between 13 and 22 years. Learners older than 18 years were excluded from the analysis and the remaining children were therefore not all at the same stage of physical development, with resulting effects on their body composition. Not all recruited children gave consent for taking blood samples, so the sample size was relatively small when the groups of children were compared.

## Conclusion

This study showed a statistically significant association between serum CRP levels and physical activity in the girls, and an inverse correlation between serum CRP levels and fitness in the boys. Waist circumference was a significant predictor of serum CRP levels in the girls. These associations indicate a link between body fat, physical activity and inflammation. Obesity should be discouraged in South African children by encouraging physical activity.
